# Blood-Brain Barrier Damage as the Starting Point of Leukoaraiosis Caused by Cerebral Chronic Hypoperfusion and Its Involved Mechanisms: Effect of Agrin and Aquaporin-4

**DOI:** 10.1155/2018/2321797

**Published:** 2018-02-26

**Authors:** Jing Huang, Junwen Li, Chao Feng, Xi Huang, Liping Wong, Xueyuan Liu, Zhiyu Nie, Gangming Xi

**Affiliations:** ^1^Department of Neurology, Shanghai Xuhui Central Hospital, 996 Middle Huaihai Road, Shanghai 200031, China; ^2^Department of Neurology, Yangpu Hospital, Tongji University School of Medicine, 450 Tengyue Road, Shanghai 200092, China; ^3^Department of Internal Medicine, The Fourth Affiliated Hospital, Zhejiang University School of Medicine, N1 Shangcheng Avenue, Yiwu 322000, China; ^4^Department of Medicine, The First Affiliated Hospital, Suzhou University School of Medicine, 188 Shizi Street, Suzhou 215006, China; ^5^Department of Neurology, Shanghai Tenth People's Hospital, Tongji University School of Medicine, 301 Middle Yanchang Road, Shanghai 200072, China; ^6^Department of Neurology, Tongji Hospital, Tongji University School of Medicine, 389 Village Road, Shanghai 200065, China

## Abstract

White matter lesion (WML) is popular in the patients aged over 65. Brain edema and blood-brain barrier (BBB) dysfunction due to cerebral chronic hypoperfusion (CCH) contributed to WML. Preserving astrocyte polarity is vital for BBB integrity. In our experiment, CCH model is established by bilateral carotid arteries occlusion (2VO). Leukoaraiosis was verified by fiber density stain, and brain edema was evaluated using brain water content measuring. The expressions of agrin and aquaporin-4 (AQP4) were evaluated, as well as the integrity of BBB. Astrocyte polarity was assessed by visualizing the distribution of AQP4 on astrocyte end-feet membranes. The results showed that expression of AQP4 firstly increased and then decreased, as agrin expression decreased gradually. At 3 days after 2VO, AQP4 and agrin displayed the most opposite expression with the former increasing and the latter decreasing; at the same time, brain edema reached high point as well as BBB permeability, and astrocyte polarity was degeneration. In the later phase, brain edema and BBB permeability were getting recovered, but WML was getting more evident. In accordance with that, agrin and AQP4 expression decreased significantly with astrocyte polarity reducing. We speculated that agrin and AQP4 played key roles in development of WML by mediating BBB damage in CCH, and BBB dysfunction due to reduced astrocyte polarity is the starting point of WMH.

## 1. Introduction

Cerebral white matter hyperintensities (WMH) are recognized as bright areas of high signal intensity in T2-weighted and diffusion tensor magnetic resonance imaging (MRI) [1]. In the over-65 aging brains, the incidence rate of lesions is prominent [2–4]. WMH are closely associated with cognitive impairment. For instance, WMH may slow information processing speed and impaired executive function [2, 5–8]. The exact pathogenesis of WMH is unclear. The main research directions for WMH pathogenesis refer to cerebral chronic hypoperfusion (CCH), blood-brain barrier (BBB) damage, and endothelial dysfunction [9–12].

Considerable evidences support that CCH is an important mechanism of WMH. Studies on the cerebral blood flow using various technologies, including computer tomography, MRI, and fluid-attenuated inversion recovery, show that a hypoperfusion status exists in the WMH area [13, 14]. The BBB damage may be vital in the incidence of white matter lesions. BBB is a physical barrier essential for the maintenance of brain function, in which astrocyte polarity is necessary. Astrocyte polarity means the specific aggregation of water channels and potassium in the superficial and perivascular astroglial end-feet membranes. Studies on human glioblastoma show that BBB permeability increases, which is associated with a decrease or loss of astrocyte polarity [15]. Astrocyte polarity is characterized by orthogonal arrays of intramembranous particles (OAPs) [16, 17] which are square arrays of particles that consisted of AQP4-M1 and AQP4-M23 [18]. These are two major isoforms of AQP4 localized in a polarized manner on astroglial being highly concentrated at the perivascular end-feet domains of astrocyte as symbol. The ratio of the AQP4 isoforms M1 to M23 influences the size and stability of the OAPs, and even though the functional relevance remains to be elucidated, we still realize that higher AQP4-M23 ratio means more stable OAPs [19, 20]. AQP4 controls BBB functioning, and modifying its expression or distribution may lead a consequent damage of BBB during the period of ischemia/reperfusion injury, but the exact relationship between AQP4 and BBB opening is unknown [21–23].

Evidence has been accumulating that suggests an important role for Agrin in CNS, in which dystrophin glycoprotein complex (DGC) preformed an important function. DGC is a multifunctional protein complex, including dystroglycan (DG), syntrophin, and scaffold protein dystrophin. It has been extensively investigated in muscle cells, in which it is linked to the extracellular matrix such as agrin and laminin, providing structural integrity during muscle contraction [24]. DGC is also present in the glial end-feet as a bridge to connecting extracellular matrix agrin and AQP4, and this connection between agrin and AQP4 is the important construction to secure the astrocyte polarity and integrity of BBB [25]. Furthermore, agrin, not only in vivo but also in vitro, may facilitate the clustering of AQP4 into OAPs on glial membrane [24, 26]. The absence of agrin leads to loss of polarized expression of AQP4 or in other words the formation of OAPs at the astrocyte end-feet [23]. However, the exact molecular mechanisms of the OAP formation and how agrin works have not been fully elucidated.

A CCH-induced WMH model was established in this study. The changing processes of cerebral edema and BBB permeability were observed. In the meanwhile, expression change curves of agrin and AQP4 were recorded at different time points after CCH. The potential mechanisms of CCH on the WMH process were explored.

## 2. Experimental Procedures

### 2.1. Animals and Grouping

Adult male Wistar rats (weighing 240 g to 280 g, 7 weeks of age) were obtained from Shanghai Slac Biotechnological Company (Shanghai, China). All animal protocols were reviewed and approved by the Ethical Committee for the Care and Use of Laboratory Animals at Tongji University. Experiments performed in accordance with the National Institutes of Health Guide for the Care and Use of Laboratory Animals (NIH Publications Number 80-23) revised 1996. The animals were housed in plastic cages with soft bedding and maintained at ambient temperature (22 ± 2°C) and humidity (55%  ±  10%) with a 12 h to 12 h light-dark cycle. The animals were allowed free access to food and water before the experiment.

All rats were treated based on the principles stated in relevant Chinese stipulations on animal experiments. All animals (*n* = 169) were randomly allocated to the two experimental groups: sham and model. The rats were randomly picked from cages for sham processing. A total of 27 rats were selected. The remaining rats were further divided into five subgroups for bilateral common carotid artery occlusion (2-vessel occlusion [2VO]) processing: 3-day, 1-week, 2-week, 1-month, and 3-month groups. At 3 days, 1 week, 2 weeks, 1 month, and 3 months following 2VO, five rats from each group were used to analyze the white matter change, five to determine the brain water content (BWC), five to assess BBB permeability, five to conduct Western blot analysis and real-time polymerase chain reaction (RT–PCR), and another five to perform immunofluorescence staining. No significant difference was observed between the subgroups. The number of animals in each group represented the actual usage suitable for the statistical analyses and did not include the rats that were eventually eliminated.

### 2.2. Rat Model Development

The adult male Wistar rats were induced CCH by 2VO processing, as previously described [27, 28]. Briefly, the animals were anesthetized with pentobarbital (Inactin, 50 mg/kg, ip). Bilateral common carotid arteries were separated and isolated, and one of the common carotid arteries was ligated with 3-0 sutures through a ventral median incision. After 1 week, the other artery was ligated. In the control group, the bilateral carotid arteries were manipulated as in the 2VO group but not through ligation. After surgery, the rats were allowed to recover at ambient temperatures (21°C to 23°C). The time points were set to 3 days, 1 week, 2 weeks, 1 month, and 3 months after 2VO.

### 2.3. Assessment of White Matter Change

The fiber density of Luxol fast blue- (LFB-) stained sections was evaluated to determine the severity of WMH during the chronic ischemia-produced hypoperfusion status. After 0.9% saline was perfused transcardially, followed by 4% paraformaldehyde, brains (*n* = 5 per group) were removed and postfixed for 24 h and then embedded in paraffin. The coronal sections were mounted onto slides and deparaffinised. The slides were stained with LFB/cresyl violet. Briefly, sections were placed in 1% LFB solution (Sigma Aldrich, Germany) at 60° overnight. Sections were rinsed in 95% ethanol and distilled water. Five sections were then differentiated in 0.05% lithium carbonate solution, followed by 70% ethanol twice, and then rinsed in distilled water again. Sections were counterstained with 0.25% cresyl violet solution for 45 s, placed in distilled water, and differentiated in 95% ethanol for 5 min. The demyelination of the regions of bilateral white matter areas were visualized at least 5 s each mouse under the inverted phase contrast microscope (Olympus/IX83, Tokyo, Japan).

### 2.4. Assessment of Brain Edema

Brain water content (BWC) was measured using the dry-wet method at 3 days, 1 week, 2 weeks, 1 month, and 3 months after 2VO. The rat brains (*n* = 5 each group) were removed and immediately weighed (wet weight), and then the brains were placed in a drying oven at 60°C for 72 h to obtain their dry weight. The BWC was calculated using the Blliot formula: [(wet weight − dry weight)/wet weight] × 100%.

### 2.5. Evaluation of BBB Permeability

Two hours prior to sacrifice, the rats (sham group: *n* = 5; 2VO group: *n* = 25) in each group were injected with 2% EB (4 mL/kg) (Sigma Aldrich, Germany) via the caudal vein. At 3 days, 1 week, 2 weeks, 1 month, and 3 months after 2VO, the infusion with heparinised saline through the left ventricle was performed until a colourless infusion fluid was obtained from the right atrium. The rats were then decapitated. The bilateral cerebral hemisphere was weighed, homogenised with 50% trichloroacetic acid, and centrifuged at 15,000 rpm for 20 min. The optical density (OD) value of EB was determined by an enzyme microplate reader (Perkin Elmer, USA) at 620 (excitation) and 680 nm (emission). The calculations of the EB content were based on a standard curve to assess the BBB permeability.

### 2.6. Immunofluorescence Analyses

The rat brain sections were fixed in 4% paraformaldehyde for 30 min. After the sections were blocked at room temperature for 60 min, they were incubated with the primary antibodies overnight at 4°C. Mouse monoclonal anti-AQP4 (1 : 100, Abcom, USA) and rabbit monoclonal anti-GFAP (1 : 200, DAKO, Denmark) were applied. To control for nonspecific staining or autofluorescence, PBS was used to replace the primary antibodies as a negative control. After washing, the sections were incubated with the secondary antibodies (Alexa Fluor 488 Goat Anti-Rabbit IgG and Alexa Fluor 594 Goat Anti-Mouse IgG, 1 : 200, Santa Cruz, USA) for 2 h at room temperature. To facilitate orientation, all sections were counterstained with DAPI (Beyotime, China). Images were obtained using a confocal laser scanning microscope (Olympus/FV1200, Tokyo, Japan).

### 2.7. Western Blotting Analyses

The protein expression was determined by Western blot (see Figure 5). In sham group, at 3 days, 1 week, 2 weeks, 1 month, and 3 months after 2VO, five rats in each group were decapitated. Proteins were extracted from cerebral tissues. The protein concentrations were detected by the BCA method. Protein samples were added on an 8% SDS-PAGE gel for electrophoresis and blotted onto PVDF membranes (Millipore, USA). The membranes were blocked with 10% nonfat milk in tris-buffered saline for 1 h. The PVDF membranes were separately incubated with the primary antibodies (1 : 500, rabbit monoclonal anti-agrin, Santa Cruz, USA; 1 : 1000, mouse monoclonal anti-AQP4, Abcom, USA; 1 : 3000, mouse monoclonal anti-tubulin, Sigma Aldrich, Germany; 1 : 10000) overnight. After washing, the membranes were incubated with the HRP-labelled secondary antibodies (1 : 3000, Goat Anti-Rabbit IgG; 1 : 3000, Goat Anti-Mouse IgG, Santa Cruz, USA) for 2 h at room temperature. The specific bands of agrin, AQP4, and tubulin were then imaged with LAS4000 (Image Quant LAS4000, GE, USA), and the OD of these bands was quantitatively analyzed with the QuantityOne software (Bio-Rad, USA). All expressions of target genes were measured and normalized by tubulin.

### 2.8. Real-Time PCR Analyses

The mRNA levels of agrin and AQP4 were determined by quantitative RT-PCR. Total RNA was extracted from the brain tissue based on the TRIzol kit instructions. Extracted RNA was then reverse-transcribed into cDNA using the Takara PrimeScript RT Reagent Kit (Takara, Japan). The cDNA was used as the template in real-time PCR reactions to analyze the expression of agrin and AQP4. The primers in Table 1 were used in these protocols.

The PCR was amplified using the Bio-Rad CFX96 Detection System (Bio-Rad, USA). The PCR conditions were performed in a 25 *μ*L volume system as follows: predenatured at 95°C for 5 min, denatured at 95°C for 20 s, annealed at 60°C for 20 s, and extended at 72°C for 1 min. All operations were repeated 40 times and then extended for 10 min at 72°C. The qRT-PCR products were assessed by melting curve analysis. The mRNA expressions of target genes were measured and normalized by tubulin as a housekeeping gene. The changes in gene expression were calculated using the 2^−ΔΔCt^ method, as previously described [29].

### 2.9. Statistical Analysis

All statistical analyses were performed with the SPSS 20.0 software package. Data from the same time points between different groups were compared by *t*-test, whereas data across different time points within the same group were compared with two-way ANOVA. All reported *p* values were two-sided, and a value of *p* < 0.05 was considered statistically significant.

## 3. Results

### 3.1. Effect of CCH on White Matter Change

**Figure 1:**
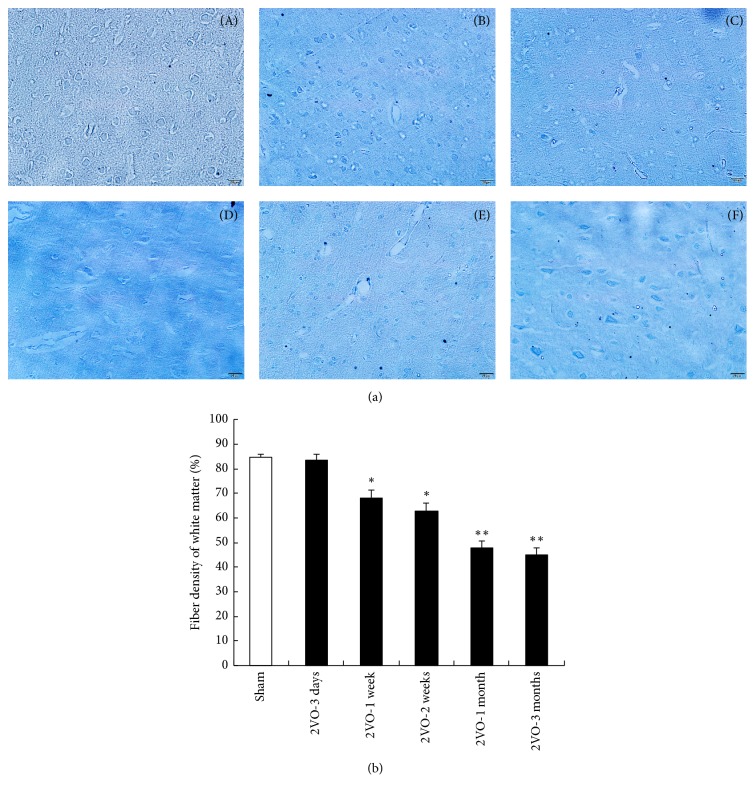
Fiber densities being stained with Luxol fast blue in the deep white matter of the CCH rats (a) and quantitative fiber densities (b). From direct microscopical observations, LFB staining was slightly affected at 3 days (B), 1 week (C), and 2 weeks (D). At 1 and 3 months after 2VO (E, F), the fiber density was significantly decreased compared with that of the sham control (A). (^*∗*^*p* < 0.05, ^*∗∗*^*p* < 0.01, compared with the brain of sham control animals; *n* = 5 in each group, LFB 200x).

LFB staining showed the changes in WMH during the chronic ischemia-produced hypoperfusion status.  Figure 1 showed that, at 3 days after 2VO, the fiber densities of the cerebral white matter of rats were similar to those of the sham group, and the fiber density was quantitatively analyzed with the Image-Pro Plus 6.0 software (Media Cybernetics, USA). At 1 week and 2 weeks after 2VO, the fiber densities began to decrease and further decreased at 1 month after 2VO. At 3 months after 2VO, the fiber densities of the cerebral white matter were significantly lower than those of sham group animals.

### 3.2. Effect of CCH on Brain Edema

**Figure 2:**
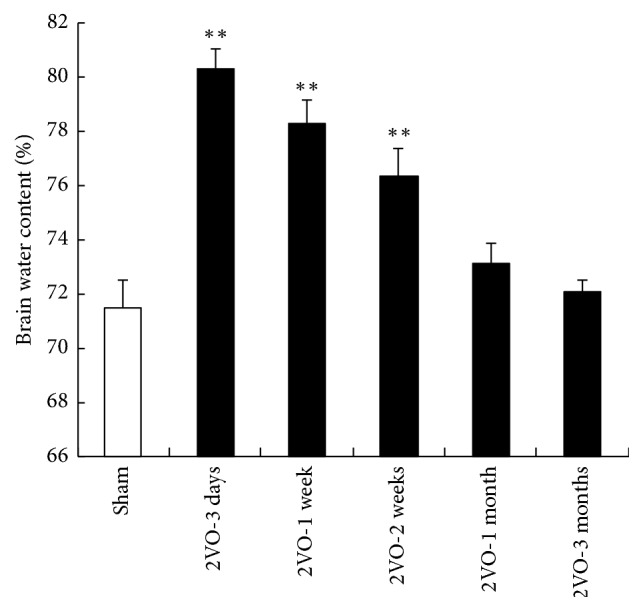
Effect of CCH on BWC. Brain tissue water content of rats was markedly increased at 3 days after 2VO, and then it had been recovering since 1 week after operation. At 3 months, BWC returned to the normal level (^*∗∗*^*p* < 0.01 compared with sham control animals brain; *n* = 5 in each group).

At day 3 after 2VO, the BWC significantly increased to reach its maximal value compared with the control rats (*p* < 0.01; Figure 2), and then the edema had been gradually recovering. At 3 months after 2VO, the BWC was still higher than that of the sham control animals even though there was no difference between these two groups.

### 3.3. Effect of CCH on BBB Permeability

**Figure 3:**
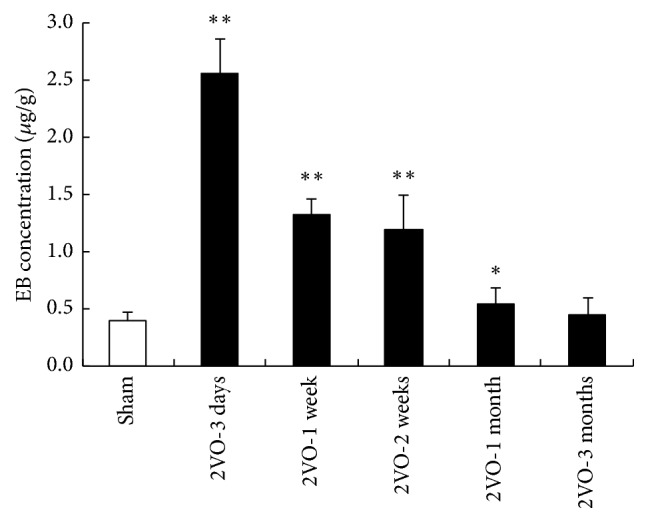
Effect of CCH on EB extravasations. Analysis of the cerebral concentration of EB. The amount of EB extravasation was increased to get the maximal point at 3 days after 2VO. And then the EB extravasation began to decrease compared with that of the 3 days. In 3-month group, the EB concentration was slightly higher than that of control group (^*∗*^*p* < 0.05, ^*∗∗*^*p* < 0.01, compared with the brain of sham control animals; *n* = 5 in each group).

The quantitative measurements of the Evans Blue dye for the operation animals revealed that, at 3 days after 2VO, the EB concentration significantly increased to get the maximal point compared with that of the sham group rats, and then it began to decrease at 1 week after operation. In 3-month group, the EB concentration was slightly higher than that of control group (Figure 3). These results demonstrated that the impairment of the BBB induced by brain CCH in the acute phase is more serious than that in the chronic phase.

### 3.4. Expression and Distribution of Agrin and AQP4

The distribution of AQP4 that was assessed by immunofluorescence changed depending on the duration time of hypoperfusion as previously described [30, 31]. The double labelling with green GFAP and red AQP4 showed that they overlapped a lot surrounding vascular in control group with brighter yellow colour as symbol. Since cerebral vascular was covered by astrocyte end-foot, we assumed that most of the yellow colour surrounding the vascular shape originated from double labelling of GFAP and red AQP4 on astrocyte end-foot. At 3 days after 2VO, the yellow colour surrounding the vascular turned lighter than that of the control group; on the other hand, yellow colour was evident on astrocytic cell body. At 1 week after 2VO group, denser yellow colour was shown on astrocytic cell body instead of mainly on end-foot before. This kind of tendency became evident with ischemia time lasting but had been becoming gentle 1 month later. It implied that AQP4 distribution had been changing from previously predominantly on astrocyte end-foot into over whole astrocyte surface since 3 days after 2VO.

**Figure 4:**
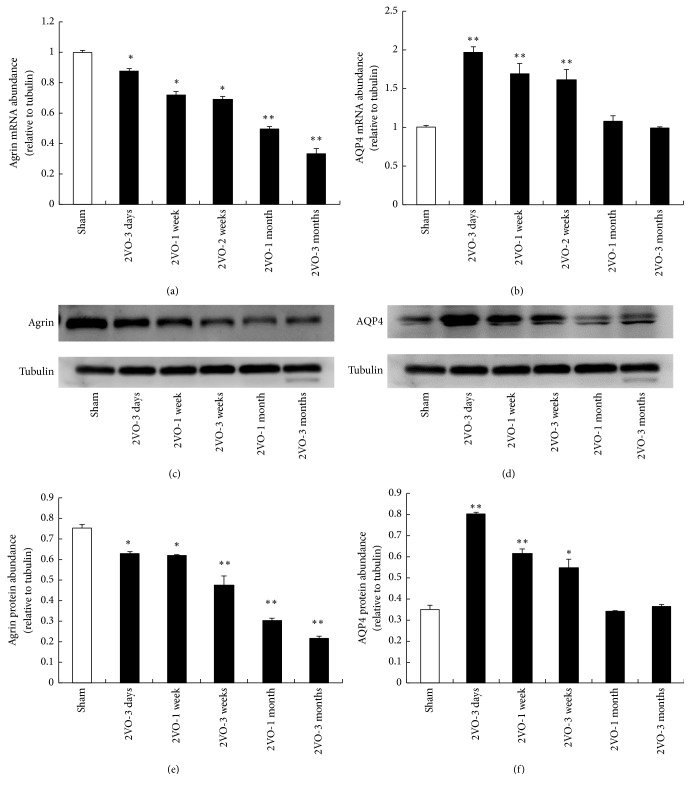
The mRNA or protein level of agrin and AQP4 after 2VO. (a) The representative picture revealed the decrease of agrin mRNA after 2VO. (b) The changing trend of AQP4 mRNA expression was bidirectional. ((c) and (d)) Representative immunoblot of agrin and AQP4. ((e) and (f)) Quantification of agrin and AQP4 protein expression. (^*∗*^*p* < 0.05 and ^*∗∗*^*p* < 0.01, compared with the brain of sham animals; *n* = 5 in each group).

**Figure 5:**
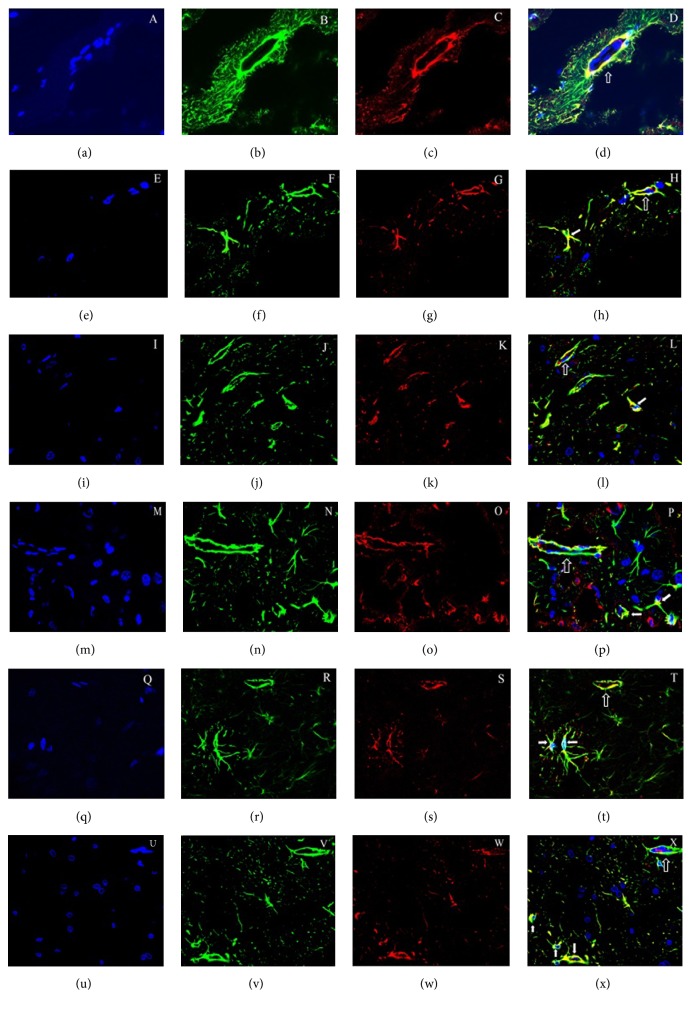
Immunofluorescence colocalization for GFAP and AQP4 in the brain. The blue nucleus in the brain was DAPI labelled. AQP4 immunohistochemistry labelling in astrocytes was red. GFAP positive astrocytes in the brain were labelled green. The coexpression of GFAP and AQP4 within the same cell is yellow (merged). Sham group (a, b, c, and d), 2VO-3 days' group (e, f, g, and h), 2VO-1-week group (I, j, k, and l), 2VO-2 weeks' group (m, n, o, and p), 2VO-1-month group (q, r, s, and t), and 2VO-3 months' group (u, v, w, and x), respectively. AQP4 was obvious on astrocyte end-feet membranes in the sham group with yellow colour as symbol, but the yellow colour had been changing from end-feet membranes to astrocytic body since 3 days after 2VO, and the alteration had been persisting even 3 months after 2VO (IF 600x).

The mRNA or protein level of agrin began to downregulate gradually after 2VO (Figure 4). At 3-month after operation, the expression of agrin reached the minimum point. On the contrary, AQP4 was upregulated significantly including mRNA and protein expression after 2VO, and it reached its maximal value at 3 days after operation and then decreased; in the 3-month group, the AQP4 expression is even lower than that of the control group. The changing trend of AQP4 expression was bidirectional, just like brain edema and BBB permeability dysfunction, different with the changing trend of agrin expression which was on-way changing curve.

## 4. Discussion

WML are considered to represent cerebral small vascular disease. Cerebral small vessel endothelial cells and smooth muscle cells were damaged in the patients who suffered from long term hypertension, which may result in the occurrence of WML. So far, many researches have been referring to the pathology of WML. Some papers proved that, despite cerebral small vascular disease, WML development and progression occur, in part, as a result of regional or entire cerebral hypoperfusion [32, 33]. Disruption of the BBB is another important mechanism implicated in the pathogenesis of WML [28, 34].

BBB integrity relies on the polarity of astrocyte which is characterized by OAPs that are square arrays of particles that consisted of AQP4 on astrocyte end-feet membranes. According to Wolburg et al., the main reason for increased BBB permeability of glioblastoma is the loss or reduction of the OAPs, which is the result of the degradation of the proteoglycan agrin [15, 33]. The heparan sulphate proteoglycan, agrin, worked as component of the extracellular matrix (ECM), is another important element for OAPs formation. Lack or decrease of agrin may cause loss of OAPs on astrocytic end-feet [24]. In our experiment, both BBB opening and brain edema got maximum point at 3 days after 2VO operation. Besides that, we found expression of AQP4 coincided bilateral curve firstly increased and then decreased, and the demarcation point was also 3 days after 2VO. At this time point AQP4 and agrin displayed the most opposite expression, as agrin expression decreased gradually with cerebral hypoperfusion lasting. Besides bilateral variation of AQP4 expression, the double labelling of green GFAP and red AQP4 performed an alteration. As we all know, AQP4 is predominantly located and concentrated in perivascular and superficial astroglial end-feet membranes to keep the polarity of astrocyte as it was shown on our control group picture, but this special distribution way had been altering since 3 days after 2VO characterized by changing its location from astrocyte end-foot to whole astrocytic body diffusely. It had clearly been shown by many researches that agrin had an effect on assembling AQP4 molecules in the membrane into OAPs, and decreased agrin expression might lead to redistribution of AQP4, which then led to BBB opening and subsequent damage [15, 23, 24]. In our experiment, the opposite expression of AQP4 and agrin might be a start symbol of astrocyte polarity disappearing. The increased AQP4 expression in the early phase may act as a compensation to save damaged BBB permeability due to decreased agrin which results in the decrease of OAPs.

Two main types of brain edema are classified as cytotoxic edema and vasogenic edema. AQP4 is involved in formation of cytotoxic edema and elimination of vasogenic edema [35]. In our findings, the AQP4 expression significantly increased in early phase after injury, which would result in cytotoxic edema, and then gradually decreased, even lower than the control level at last. We assumed that, in the early stage of cerebral ischemia, the sharp decline of cerebral blood flow led to the energy failure, which then lead to another big problem that the cells have no enough energy to draw water out of the cell. It is one of the main reasons to cause cytotoxic edema.

With continuing ischemia, the cellular damage would cause further BBB dysfunction which contributed to leakage of plasma proteins into extracellular space. In our experiment, at 3 months after 2VO, although agrin and AQP4 expression were still lower than those of the control group, the trend had been retarding when compared with 1-month group, and increased BWC due to BBB permeability had been recovering as well, The mechanisms of brain edema recovery during the chronic hypoperfusion period were complex, including recruitment of nonperfused capillaries, angiogenesis, and biochemical regulation of the CBF, as well as an enhanced immunocytochemical signal for vascular endothelial growth factor (VEGF) [36] and matrix metalloproteinases (MMPs) [37] and action of other agents such as reverse pinocytosis [38] and disputed Ca2+ signalling [39]. So we guess, in the late stage of cerebral ischemic, the reasons of edema alleviation were complex more than just AQP4 [40, 41].

The similar variation curve between BBB opening and brain edema implied that brain edema may be the result of BBB opening. At early stage the WMH was insignificant. However, despite EB leakage from vessels was getting recovered in the later stage, the WMH began to appear gradually. We assumed that the BBB opening may cause not only brain edema, but also leakage of elements from serum, which may be a starting factor that initiated damage of WM ultimately.

We speculate that BBB dysfunction is the starting point of WML after 2VO, and agrin played key role in preserving BBB function; in our experiment, decreased astrocyte polarity due to downregulation of agrin is responsible for BBB opening and WML progress. However, in our study neither a direct correlation between the loss of AQP4 and behaviour of OAP phenotype nor AQP4 isoform depression ratio was elucidated. Additional studies are needed to observe all the follow-up changes after 3 months including subsequent variation characteristic of agrin and AQP4, as well as other possible agrin and AQP4 related mechanisms for white matter lesions [42], and it may help us to know more about how ingenious brain worked to adapt the hypoperfusion so as to target them to relieve the white matter lesions.

## Figures and Tables

**Table 1:** Primer sequences for quantitative RT–PCR.

Gene	Primer	
	Forward	Reverse
Agrin	AGAAGAACAAGTTGCCATGG	ACGGATGGTCTCCACATTCT
AQP4	TTGCTTTGGACTCAGCAT TG	GGGAGGTGTGACCAGGTAGA
Tubulin	TGAGGCCTCCTCTCACAAGT	CGCACGACATCTAGGACTGA
